# A Rare Case Report of Neurological Condition: Moersch-Woltman Syndrome with Positive Anti-GAD Antibodies

**Published:** 2023-09-15

**Authors:** Aakanksha Pitliya

**Affiliations:** 1 MBBS, Extern, Neurology Services Inc, Alexandria, Virginia, USA

## Abstract

**Background:**

Moersch-Woltman Syndrome (MWS), also known as Stiff person syndrome (SPS), is a rare, progressive condition of the central nervous system. Symptoms can include severe immobility, rigidity, and painful muscle spasms in the trunk and limbs. Muscle spasms can occur in MWS patients because they are more sensitive to noise, rapid movements, and mental disturbance. The condition affects women twice as frequently as it does males. It is a rare disease, affecting only 1-2 people per million. The case report aims to highlight the importance of the diagnostic challenges associated with MWS and the significance of glutamic acid decarboxylase (GAD) antibodies.

**The case:**

A 57-year-old female patient presents with history of migraine headaches, anxiety, and depression. The patient experienced widespread and distressing muscle spasms affecting the shoulders, upper and lower back, and limited range of motion in the neck. Physical examination revealed dense diffuse muscle stiffness throughout the body. Further investigations were ordered, including a comprehensive range of laboratory tests and imaging tests. A positive test for GAD antibodies confirmed the diagnosis of MWS. Treatment included administration of Clonazepam and Baclofen. A follow-up appointment, three weeks later, indicated a noticeable 15-20% reduction in spasticity.

**Conclusion:**

This case highlights the crucial role of anti-GAD antibodies in confirming the diagnosis of SPS. Healthcare professionals should consider testing for these antibodies in patients presenting with the described symptoms. A multidisciplinary approach involving neurologists, physical therapists, psychiatrists, and orthopedic surgeons is essential to provide comprehensive care and optimizing outcomes for individuals with MWS.

## Introduction

Moersch-Woltman Syndrome (MWS), also known as Stiff person syndrome (SPS), is a rare, progressive condition causing increased muscular activity brought on by a decline in brain and spinal cord inhibition. It manifests as gradual muscle rigidity, usually in the axial muscles. However, rigidity can also occur in the extremities. Besides rigidity, the patient frequently experiences strong, sporadic muscle spasms that may be spurred by jarring noises, bright lights, or mental distress.^1^ Although more recent studies have found several other antibodies associated with the disease, including antibodies against GlyR, GlyR-associated protein (GABARAP), and GlyT2, patients with MWS are most frequently positive for antibodies against glutamic acid decarboxylase (GAD 65).^2^ Confirming the diagnosis of MWS can be challenging, as antibodies against GAD are most frequently reported and are positive in only 60-80% of patients.^2,3^ However, they are beneficial in confirming MWS when combined with the clinical picture.^2^

The quality of life of a patient is substantially impacted by MWS, which is also associated with neurological and psychiatric diseases.^4^ There is evidence of genetic influence on the risk of developing MWS; the HLA class 2 locus makes patients susceptible to the condition.^5^ Unfortunately, the diagnosis is often delayed, with an average of six years passing from symptom onset to diagnosis.^5^ It is reported that diazepam and other benzodiazepines, corticosteroids, plasma exchange, and intravenous immunoglobulin have been effective in improving symptoms of the disease, but none alter the progression of the disease.^6^

The cause of this condition is rarely known, making it difficult to control disease progression. The reason behind GAD autoimmunity manifestation in MWS patients is unknown, and it is debatable whether MWS meets the criteria for classification as a neuro-autoimmune condition. It is also questionable whether these antibodies are pathogenic since the levels of antibody titers do not correlate to the severity of the condition.^7^ It has not been established that GAD antibodies are the only cause of MWS, and the possibility of GAD acting as a biomarker for the disease is doubtful.^7^ There is no evidence-based criterion for treating MWS, and the rarity of the disease complicates the efforts to establish guidelines.^6^

We present the case of a 57-year-old woman with a history of migraine headaches, anxiety, and depression who developed diffuse painful muscle spasms in her shoulders, upper and lower back, and inability to move her neck. Dense diffuse muscle stiffness was found throughout the body on examination. In addition to the clinical presentation, the diagnosis of MWS was confirmed by positive GAD antibodies.

## Case

A 57-year-old woman presented to the neurology clinic with chief complaints of diffuse muscle stiffness, pain, and spasms in her head, neck, and back, persisting and slowly progressing for the past 15-20 years. These chronic and progressive symptoms have significantly impacted her activities of daily living, worsened by inadequate sleep but relieved by sleep and exercise.

During her time in the Air Force from 1980 to 1999, she experienced milder symptoms that have since worsened. She recalls a head injury in the 1990s without associated weakness. Her current symptoms include stiffness, bilateral arm numbness and tingling, burning fingertips, gluteal numbness with prolonged sitting, muscle twitches without fixed position, spine stiffness, balance loss, diffuse muscle spasms, and widespread pricking pain. She also experiences sleep difficulties, bending and turning issues, and autonomic symptoms like urinary urgency and constipation. Prolonged postures worsen her pain, and medications provide little relief. Her condition has impacted her intimate relationship.

The patient has known allergies to fish product derivatives, iodine-containing contrast media, porcine derivatives, shellfish, and erythromycin. Her mother had a history of colon cancer and osteoarthritis. She plans to retire early due to her condition, but her supervisors are unaware and unsympathetic. She experiences chronic pain that has worsened with age. She has a past medical history of migraine headaches, anxiety, and depression.

She takes multiple medications for her symptoms, including Albuterol, Budesonide-Formoterol, Eetirizine, Epinephrine, Famotidine, Fluticasone Propionate, fovatriptan, Gabapentin, Ibuprofen, Lisinopril, Magnesium oxide, Naproxen, Ondansetron, Pantoprazole, Polyethylene glycol, Polyvinyl alcohol, Ropivacaine, Triamcinolone, and Zolmitriptan.

During the examination, she appeared stiff and moved rigidly without trunk twisting. She preferred standing due to stiffness. Weakness in her arms and legs, difficulty lifting her leg while walking, and occasional foot dragging were noted. Simple tasks are challenging due to poor hand grip, as tested. She perceives weakness as peripheral. Physical therapy has become more difficult, but she remains committed to exercising.

Neurological examination revealed spasms in the shoulders and back, limb muscle stiffness, normal reflexes, decreased distal muscle strength, 3/5 graded muscle tone, and broad-based gait. The ocular examination was normal, and the rest of the neurological examination was unremarkable.

Additional investigations included laboratory tests, MRI scan that revealed no abnormalities, and planned EMG and NCS. Notably, the patient’s GAD antibody test confirmed a positive result, further supporting the diagnosis of MWS. The treatment regimen involves the administration of Clonazepam and Baclofen. At a 3-week follow-up, she reported 15-20% relief in spasms and improved daily living activities. Intravenous immunoglobulin therapy for quick relief is scheduled. After two months, she reported a 30-40% improvement in spasticity and activities of daily living.

## Discussion

MWS is a rare disorder of the central nervous system characterized by rigidity and stimulus-triggered painful muscle spasms of predominantly axial and proximal limb muscles.^2^ The differential diagnosis for MWS is broad, including disorders of the brain, spinal cord, and muscles such as myelopathies.^7^ Table 1 summarizes the differential diagnosis of Moersch-Woltman Syndrome.

Even though the patient exhibited several symptoms that were quite typical for MWS, her co-morbid illnesses likely contributed to the delay in diagnosis. She had a long history of depression, anxiety, and migraines, to name a few. It does not seem unreasonable to suppose that her psychiatric history may have played some role in her presentation. However, it is crucial to remember that MWS and concomitant autoimmune disorders frequently cause anxiety.^4,8^

MWS patients generally have GAD antibodies, which seldom occur in the general population.^9^ In addition to blood tests for GAD, an electromyography test can help confirm the condition’s presence as it generally reveals continuous agonist and antagonist muscle motor activities.^7,9^ Benzodiazepine-class drugs are the most common treatment for symptom relief from stiffness.^10^ Intravenous Immunoglobulin and plasmapheresis, among other immunotherapies, may also be prescribed.^10^

The prevailing and logical approach to therapy involves a combination of GABA-enhancing medications and immunotherapy. This is because these two treatment categories operate through distinct mechanisms: one addresses underlying pathological processes, while the other targets autoimmune responses.^11^ Dalakas et al. (2023) recommended initiating antibody treatment without any delay, concurrently with the administration of antispasmodic medications.^12^ The patient’s condition was improved using a variety of treatments after the diagnosis. Diazepam, a GABA-enhancing medication, and Baclofen, an antispastic medicine, were first administered to the patient. According to Dalakas et al. (2009) and Dalakas et al. (2023), both are routinely suggested as possibilities for the first treatment.^12,13^ Although there is currently no treatment that cures MWS, working with a specialist and maintaining symptom control can make it easier to live with the condition.^14^

### Strengths

In approaching this case, several strengths can be identified. Firstly, a thorough review of the relevant medical literature on MWS and its differential diagnosis was conducted, providing a comprehensive understanding of the condition and its diagnostic challenges. This allowed for a more informed assessment of the patient’s symptoms and formulating a differential diagnosis. Additionally, the inclusion of relevant diagnostic tests, such as blood tests for GAD antibodies and electromyography, contributed to the confirmation of the MWS diagnosis.

### Limitations

This case report represents a single patient, which limits the generalizability of the findings to a larger population. Further studies involving larger sample sizes are needed to corroborate these observations. Additionally, the absence of long-term follow-up data restricts the assessment of treatment outcomes and prognosis.

### Conclusion

The differential diagnosis for MWS is broad, highlighting the importance of thorough evaluation and ruling out other potential conditions. Further research and awareness are needed to enhance understanding and facilitate the development of more effective treatments for this challenging condition.

By recognizing the potential presence of MWS, accurate diagnoses can be made, leading to appropriate management and treatment strategies. This emphasizes the importance of maintaining a high index of suspicion for MWS to improve patient outcomes and prevent unnecessary delays in the appropriate management of this condition.

## Summary – Accelerating Translation

The rare and progressive disorder Moersch-Woltman Syndrome (MWS), also known as Stiff Person Syndrome (SPS), is characterized by muscle rigidity and spasms and has a significant impact on a person’s quality of life. This case report focused on shedding light on the difficulties in detecting and managing this complicated condition by examining the clinical presentation, diagnosis, and treatment of MWS. The study involved a comprehensive evaluation of a 57-year-old woman presenting with chronic muscle stiffness, pain, and spasms. Based on the patient’s clinical condition and the presence of GAD antibody positivity, the diagnosis of MWS was established. Treatment options such as Clonazepam and Baclofen were administered, and the patient’s response was evaluated. The patient presented signs of MWS, such as tight muscles, spasms, and sensory problems. The diagnosis was supported by laboratory studies that revealed the existence of GAD antibodies. A partial improvement in the patient’s spasticity and activities of daily living was seen after treatment with clonazepam and baclofen. MWS, a challenging neurological condition, manifests as crippling muscle rigidity and spasms. Because of its rarity and the lack of clear diagnostic markers, MWS diagnosis can be challenging. However, a comprehensive examination that includes clinical assessment and pertinent laboratory tests, including GAD antibody testing, can help to support the diagnosis. Although there is no cure, treatment plans attempt to reduce symptoms and enhance the patient’s quality of life. Further research and awareness are needed to enhance our understanding of MWS and develop more effective treatments.

This case report emphasizes the importance of early MWS diagnosis and treatment, as well as the necessity of multidisciplinary management involving neurologists, physical therapists, and other experts. Healthcare practitioners can better support people with this difficult condition, improving their outcomes and general well-being, by raising awareness about MWS.

## Figures and Tables

**Table 1.** Summary of the differential diagnosis of Moersch-Woltman Syndrome.^7^

Serial number	Diagnosis	Differentiating Presentation	Diagnostic tests	Comparison with MWS
1.	Myelopathies	Upper motor neuron, lower motor neuron signs, sensory deficits	MRI confirms the diagnosis.	In MWS, MRI is normal.
2.	Dystonias	Variable abnormal posturing, significant muscle pain, and cramping.	(EMG shows pulsating nerve signals being transmitted to the muscles even at rest.	EMG shows continuous motor unit activity in agonist and antagonist muscles in MWS.
3.	Spinocerebellar Ataxia	Hypermetric, slow saccades, nystagmus, areflexia, tremors, intellectual disability	Genetic testing confirms the diagnosis.	No genetic testing is required. Preserved intelligence in MWS.
4.	Primary Lateral Sclerosis	Onset after 50 years of age, Spasticity, Hyperreflexia, Babinski sign positive.	Pringle’s Criteria for diagnosis.^13^	Anti-GAD for diagnosis of MWS.
5.	Neuromyotonia	Hyperhidrosis, muscle fasciculations, quivering of the muscle, myoclonic jerks, and myotonia-like symptoms	Fibrillation potentials and fasciculations on EMG.	EMG shows continuous motor unit activity in agonist and antagonist muscles in MWS.
6.	Multiple Sclerosis	Off and on UMN symptoms, Temperature sensitivity, Optic neuritis	MRI for diagnosis	MRI normal in MWS, no temperature sensitivity
7.	Parkinson’s disease	Tremor, Rigidity, Akinesia	Clinical, DaT scan, MRI	DaT Scan is normal, no tremors are present in MWS.

MRI: Magnetic Resource Imaging, MWS: Moersch-Woltman Syndrome, DaT: Dopamine transporter, EMG: Electromyography, GAD: Glutamic Acid Decarboxylase; UMN: Upper Motor Neuron.

## Highlights:

- This case report sheds light on the clinical manifestations of MWS.
- The case emphasizes the significance of considering MWS as a differential diagnosis in patients presenting with widespread muscle spasms, even in the absence of obvious abnormalities in imaging studies.
- The confirmation of GAD antibodies, highlights their crucial role in diagnosing Stiff-Person Syndrome and emphasizes the importance of testing for these antibodies in patients with muscle stiffness and spasms
- The successful reduction in spasticity with the administration of Clonazepam and Baclofen highlights the potential effectiveness of these medications in managing symptoms associated with MWS.
- The case underscores the importance of a multidisciplinary approach involving various healthcare professionals to provide comprehensive care and optimize outcomes for individuals living with MWS.
